# Best Practices to Implement Dried Blood Spot Sampling for Therapeutic Drug Monitoring in Clinical Practice

**DOI:** 10.1097/FTD.0000000000000994

**Published:** 2022-05-23

**Authors:** Marith I. Francke, Laura E. J. Peeters, Dennis A. Hesselink, Sanne M. Kloosterboer, Birgit C. P. Koch, Herman Veenhof, Brenda C. M. de Winter

**Affiliations:** * Department of Hospital Pharmacy, Erasmus MC, University Medical Center Rotterdam;; † Division of Nephrology and Transplantation, Department of Internal Medicine, Erasmus MC, University Medical Center Rotterdam;; § Division of Vascular Medicine, Department of Internal Medicine, Erasmus MC, University Medical Center Rotterdam;; ¶ Department of Child- and Adolescent Psychiatry/Psychology, Erasmus MC Sophia Children's Hospital, University Medical Center Rotterdam, Rotterdam; and; ‖ Department of Clinical Pharmacy and Pharmacology, University Medical Center Groningen, University of Groningen, Groningen, the Netherlands.

**Keywords:** dried blood spot, therapeutic drug monitoring, adherence, home sampling

## Abstract

Sampling of blood at home to determine the concentration of drugs or other compounds can be effective in limiting hospital-based sampling. This could lower hospital visits and patient burden, improve the quality of life, and reduce health care costs. Dried blood spot (DBS) microsampling is often used for this purpose, wherein capillary blood, obtained by pricking the heel or finger, is used to measure different analytes. Although DBS has several advantages over venous blood sampling, it is not routinely implemented in clinical practice. To facilitate the bench to bedside transition, it is important to be aware of certain challenges that need to be considered and addressed.

**Results:**

Here, important considerations regarding the implementation of DBS in clinical practice, the choice of patients, blood sampling, transport, and laboratory analysis are discussed. In addition, we share our experience and provide suggestions on how to deal with these problems in a clinical setting.

## INTRODUCTION

At-home blood sampling for analyte detection has the potential to lower patient burden and health care costs.^1,2^ The dried blood spot (DBS) microsampling method is frequently used in this regard; it uses capillary blood obtained by a heel or fingerprick.^3^ This method is mostly known for its use in neonates to screen for metabolic diseases, but recent studies have demonstrated the value of DBS in both children and adults, including monitoring of drug adherence and performing therapeutic drug monitoring (TDM).^3–5^ At present, different microsampling methods exist, including volumetric and nonvolumetric methods.^6^ We use a nonvolumetric DBS microsampling method at our institutions because this method is cheaper, user-friendly, and the hematocrit can be easily measured using near-infrared spectroscopy to correct for its potential effect on the measurement of an analyte.^7^ With this method, a drop of blood, obtained by a simple fingerprick, is spotted on a filter paper, from which many analytes can be determined.^3^ DBS microsampling has several advantages over venous blood sampling.^4^ It is minimally invasive and requires only a small amount of blood. Furthermore, DBS sampling can be easily performed at the clinic, but home sampling is also possible. The latter can reduce hospital visits and enable the exact measurement of predose concentrations and area under the curves, which is important for TDM.^2^ Specific guidelines have been developed for the analytical and clinical validation of DBS microsampling to improve the reliability of the DBS measurements.^6^ Despite these qualitative improvements in the validation process, only a few DBS methods are currently used in clinical practice and in the home-setting.^8^ To date, the factors underlying this discrepancy have not been clearly defined. We suggest that this gap is due to several factors that require careful attention before implementing self-sampling of patients using DBS. These factors include the choice of the patient population, clinical usefulness, instructions on the correct collection of a DBS blood sample, transport of DBS cards, and laboratory analysis.^9^ Based on our own experience of implementing home sampling, we propose some practical solutions and suggestions to expand the use of DBS sampling in clinical practice.

## PATIENT POPULATION

Although DBS sampling can be useful for many patients, it should be noted that this method is not suitable for everyone. Important considerations when implementing this method in clinical practice include preference and ability of the patient to perform DBS sampling at home and its clinical usefulness.^2^

The preference and ability of the patient to perform DBS sampling are being investigated in several ongoing studies at our tertiary-care hospital. In these studies, the pain associated with the DBS fingerprick was documented because pain can affect the willingness of the patient to undertake this sampling method. In general, patients believe that a fingerprick was not very painful. In a study including 175 recipients of solid organ transplants, the patients were asked to rate the pain experienced by them during venipuncture and a fingerprick (scale 0–10) (unpublished data, VIDA, Dutch Trial Register NL8502). The median pain score of the venipuncture [1.0; interquartile range (IQR) 0.0–3.0; range 0.0–9.0] was comparable with the median pain score of fingerprick (1.0; IQR 0.0–2.0; range 0.0–5.0). Although most patients preferred a fingerprick over a venipuncture (n = 88; 50.3%), some preferred venipuncture over a fingerprick (n = 16; 9.1%), whereas others had no preference (n = 70; 40.0%). The reasons for patients' preference for venipuncture over a fingerprick were (1) inability or unwillingness to perform blood sampling at home and (2) requirement of tests that cannot be performed with DBS sampling.

These results are in line with observations from patients with resistant hypertension who participated in a randomized controlled trial to improve nonadherence to antihypertensive drugs (unpublished data; RHYME-RCT, Dutch Trial Register NL6736). The median pain score for a fingerprick in these patients was 1.0 (IQR 0.0–2.5; range 0.0–7.0). In addition, the patients were asked whether they would be able to perform DBS sampling at home. Of the 18 patients interviewed, 4 (22%) indicated that they would not be able to perform DBS sampling at home. A study by Kloosterboer et al showed that the pain scores in children, as rated by the children themselves, were slightly higher than those observed in adults, with a median NRS-11 pain score of 2.^10^ However, the spread of pain scores was quite broad, ranging from 0 to 10 (IQR 0–7). The pain experienced by children was independent of age, sex, and identity of the person performing the fingerprick (ie, parent, researcher, or self). This study was performed on children with autism spectrum disorders and behavioral problems, which might have contributed to the fact that 1 of 5 children refused one or more fingerpricks during this study. However, most children, even in this challenging population, tolerated repeated fingerpricks very well. Finally, it is important to consider the clinical usefulness of DBS in a patient population. Patients would especially benefit from DBS sampling if they require little to no other clinical monitoring. For these patients, hospital visits could be reduced by DBS sampling, resulting in a lower burden and cost for the patient, and an increased willingness to perform DBS sampling.^2,11^ This is mainly because of fewer hospital visits and DBS sampling being cheaper than conventional venipunctures.^11^

**FIGURE 1.**
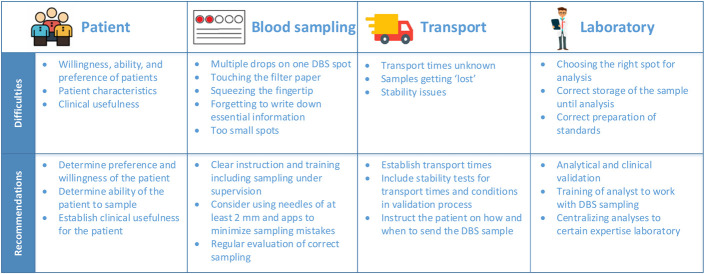
Practical considerations for implementing DBS sampling in clinical practice.

Thus, the patients' experience to DBS sampling depends on their background and characteristics (Fig. 1). The same holds true for their ability and willingness to perform DBS sampling. Therefore, one should consider all the relevant advantages and disadvantages of using DBS sampling for at-home blood sampling for each patient, thereby considering the patients' preferences, the ability and willingness to perform the blood sampling at home, and its clinical usefulness.

## BLOOD SAMPLING INSTRUCTIONS

When a patient is willing to perform DBS sampling, it is important to provide the right instructions and training to reduce the risk of patients making mistakes while collecting their own blood samples. Incorrect DBS sampling can affect the validity of the results or lead to delays because resampling is required and, therefore, should be avoided.^12^ Depending on the training provided to the patients and health care workers, 4%–58% of the DBS samples were rejected for analysis because of poor sample quality.^2,12,13^

**FIGURE 2.**
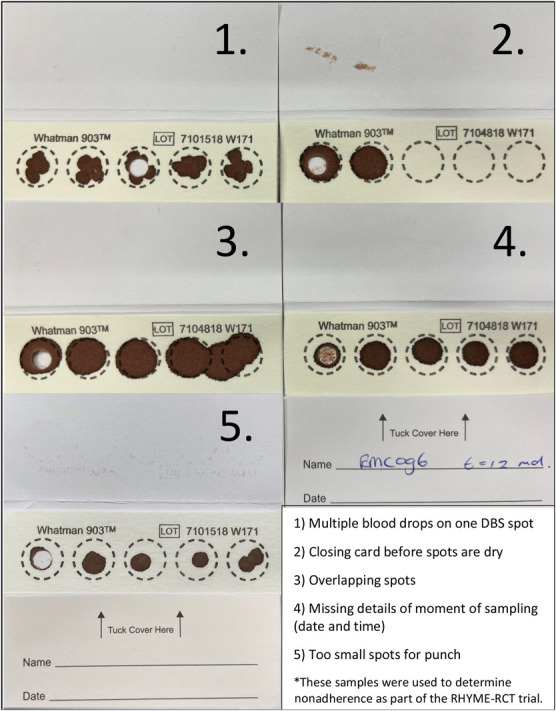
Common sampling mistakes* while using DBS sampling for measuring drug concentrations or other compounds.

In our research groups, several instructional approaches were evaluated with different populations, including video instructions, written and oral instructions, and training the patients by performing at least 1 DBS sampling under the supervision of a trained health care provider. Common sampling errors were (1) multiple drops of blood on 1 DBS spot; (2) touching the filter paper, leading to contamination; (3) squeezing the fingertip, which may dilute the blood with wound fluids; (4) forgetting to write down patient details or details of the time of sampling; and (5) spots being too small for analysis (Fig. 2). Similar mistakes in blood sampling have been previously reported as causes of rejection of DBS samples for further analysis.^13^

Adequate instructions are also important when delegating sampling to other health care providers. This can be useful when patients are admitted to the hospital for instance after transplantation. In the adherence trial, RHYME-RCT, verbal and written sampling instructions were given to the patients by health care providers instead of the researchers. However, the written instructions still resulted in problems such as small spots, irregular spots, and overlapping spots, which suggest that instructions should be provided by the “see one, do one, teach one” principle to train health care providers.

Finally, the instructions for DBS sampling in children involve additional challenges because both the child and the guardian need to be instructed on how to perform the fingerprick. Instructions for the child need to be at the right educational level to avoid resentment to the fingerprick, for example, using images instead of text. Personal instruction, in combination with test sampling, resulted in optimal participation and sampling.^10^

There are several options for assessing and improving the spot quality. First, if small spots are detected regularly, even after optimal instructions, lancets with a larger needle can be a solution. In general, we recommend using lancets with a needle length of at least 2.0 mm, even in children. Second, we recommend using 2 preprinted concentric circles in which the inner circle should be filled, giving patients the possibility to check whether the volume of the sample is enough for analysis.^14^ Finally, patients can use tools such as a web-based application, as presented by Veenhof et al, ^12^ to assess both the size and shape of the blood spot. This can decrease the number of DBS samples that are rejected for analysis and require resampling. However, it is important to realize that not all errors can be recognized by a web-based application or are visible on the DBS card.

Depending on the purpose of DBS sampling, the importance of prevention and detection of mistakes in blood sampling varies. For a qualitative analysis, for example, to determine drug adherence, the precision and shape of the spot may be less important. For a quantitative analysis, in which determination of the exact concentration of an analyte is necessary, every sampling error will lead to small deviations in the concentration measured. In addition, information about the date and time when the last dose was ingested by the patient, before DBS sampling, is especially important for the interpretation of the results in TDM. Together, these results demonstrate the importance of clear instructions, which should be adapted to individual patients, guardians, or health care providers (Fig. 1). We recommend helping the patient practice how to correctly perform DBS blood sampling. Furthermore, checking and evaluating spot quality in the laboratory is mandatory. One must intervene when the quality is suboptimal and repeat the instructions to the patients.

## DISTRIBUTION AND TRANSPORT

DBS filter cards should be distributed to patients when DBS sampling is used in clinical practice. There are several options for distributing filter cards; one can send the DBS filter cards by regular mail to the patients or to different clinics within the country. A second option is to provide the patients with DBS filter cards when they are discharged from the hospital or during their visit to the outpatient clinic. Finally, the DBS filter cards can be distributed to the patients through the pharmacy by providing patients with a combined prescription for DBS filter cards along with the drugs. The best distribution strategy depends on the patient population and where the patient is being treated (eg, university medical center versus general practitioner), the analyte to be measured (drugs versus other analytes), and how the health care and postal systems are organized in the country where the DBS method is to be implemented.

After the patient has received the DBS card and performed blood sampling at home, the sample has to be transported to the laboratory. A DBS card can be sent to the laboratory using regular mail. Depending on the postal system of the country, the hospital involved, and patient instructions, it can take some time before the sample reaches the hospital, and in the worst case, the sample never reaches the hospital. This is essential to know beforehand because doctors usually check a patient's laboratory results before the patient visits the outpatient clinic or attends a phone appointment.

We evaluated the time interval between mailing an envelope with a DBS card and arrival at the hospital laboratory by sending both normal and medical envelopes from different locations in the Netherlands. We found transport times ranging from 2 to 6 days from the time of placing the envelope in a mailbox until arrival at the hospital laboratory for analysis. The time taken for the sample to arrive at the laboratory was highly dependent on the day the DBS card was mailed (weekday versus weekend), but the type of envelope (normal versus medical) had no impact. These transport times were in accordance with a study by Veenhof et al^2^ in the Netherlands, where patients were responsible for sending the envelope back to the hospital. Although the transport times were similar, only 20% of the DBS samples were analyzed on time, that is, before the patients' appointment with the doctor. Therefore, there is a need to instruct the patients on when to send the DBS samples. To minimize transport time, reminders could be sent to the patient and a track-and-trace system could alert health care providers as to the status of the sample.^2,15^

In addition, transport time and conditions must be taken into account during stability testing, as described in the guidelines on alternative sampling.^6^ To minimize humidity problems during transportation, it is recommended that the sample is sent in a plastic bag with silica as a desiccant. Furthermore, it could be helpful to send the samples in envelopes from a mail service perspective. Usually, these service points are inside a building, which would reduce variations in temperature and other environmental conditions. Another approach to optimize transportation is to use courier services. This can shorten delivery times but also result in higher delivery costs. A futuristic solution to minimize transport time is by using drones. Although the results of the first few studies on this topic were promising, they also revealed some problems with transport conditions, and the costs were still higher than transportation using ground vehicles.^16,17^ In conclusion, before implementing home sampling, the transport times of DBS cards to the laboratory should be known. The exact transport time is usually a black box and can only be established by test-sending envelopes. Furthermore, it could be beneficial to involve the post office of the laboratory in the logistic process to improve transportation time. In addition to the transport time, stability testing during the validation process should not only include the maximal transport times but also extreme conditions that can occur during the transport of the sample.

## LABORATORY

The last challenge of DBS sampling occurs in the laboratory where the DBS sample is analyzed.

When the sample arrives at the hospital, the sample must be stored correctly until further analysis. This is usually determined during the analytical validation by stability analysis and includes storage in a desiccator or at room temperature.^6,18–20^ However, previous research has shown that analytes are more stable in DBS than in frozen plasma.^21^ The widespread use of DBS sampling can be useful to measure more samples at once and provide measurements on a regular basis. This reduces the storage time and thereby lowers the risk of analyte degradation.

Second, the choice of the most optimal blood spot for analysis is important to reduce variability in measurements. Critical evaluation of the spot before punching out the spot is, therefore, crucial for correct measurement. Because small spots are commonly seen when patients are sampled at home, a small punch diameter could increase the acceptance rate of DBS samples. However, a larger diameter is sometimes necessary to reach the lower limit of detection for a particular analytical technique.

Furthermore, to reduce the sample preparation time, an automatic puncher or fully automated DBS analyzers can be helpful.^22,23^ However, when punching out small spots that are within the diameter limits, a manual puncher can provide a more precise punch. Finally, the preparation of standards can influence the analyte concentrations measured in the samples. We found that the use of freeze-dried blood may affect the spread of blood on filter paper, resulting in lower standard concentrations. Therefore, we recommend the use of fresh blood spiked with the component(s) of interest during DBS sampling method development and analysis (unpublished data).

Because DBS sampling and analysis are relatively new in clinical practice, only a few laboratories and technicians have sufficient experience and expertise to correctly process DBS samples. Therefore, we recommend centralizing all DBS analyses to a few laboratories with significant expertise (Fig. 1). In addition, adequately training technicians, laboratory specialists, and researchers are essential to correctly process the DBS sample and accurately determine drug concentrations.

## CONCLUSIONS

DBS sampling can be a reliable and relatively easy-to-use method for home sampling in clinical practice. However, the feasibility of its implementation largely depends on the choice of the patient population, accurate instructions on blood sampling, optimal transport, and analytical experience in the laboratory, all of which should be taken into account before clinical translation.
